# Playful Testing of Executive Functions with Yellow-Red: Tablet-Based Battery for Children between 6 and 11

**DOI:** 10.3390/jintelligence10040125

**Published:** 2022-12-14

**Authors:** Ricardo Rosas, Victoria Espinoza, Camila Martínez, Catalina Santa-Cruz

**Affiliations:** 1 Centro de Desarrollo de Tecnologías de Inclusión, Escuela de Psicología, Pontificia Universidad Católica de Chile, Santiago 7820436, Chile; 2 Centro de Justicia Educacional, Facultad de Educación, Pontificia Universidad Católica de Chile, Santiago 7820436, Chile

**Keywords:** executive functions, technology-based assessment, cognitive assessment

## Abstract

Executive functions are psychological processes of great importance for proper functioning in various areas of human development, including academic performance. For this reason, from both clinical and educational perspectives, there is great interest in how they are assessed. This article describes the development and standardization process of Yellow-Red, an instrument for directly assessing executive functions in children between 6 and 11 years of age in a playful format using digital support. The test was based on a three-factor model of executive functioning: inhibition, working memory, and cognitive flexibility. Yellow-Red comprises six subtests: cognitive inhibition, behavioral inhibition, auditory working memory, visual working memory, cognitive flexibility, and a global assessment test of executive functions. The test was administered to 245 boys and girls between 6 and 11 years of age. Along with the Yellow-Red subtests, gold standard tests were applied for each of the executive functions assessed. The test’s psychometric properties are powerful in both reliability and validity evidence. The reliability indices are all greater than 0.8. As evidence of convergent validity, correlations were established between the tests, and the tests considered gold standards. All correlations were significant, with values ranging between 0.42 and 0.73. On the other hand, the factor structure of the test was analyzed using confirmatory factor analysis. Although it is possible to demonstrate the progressive differentiation of the factor structure with age, it was only possible to find two factors at older ages, one for inhibition/flexibility and one for working memory.

## 1. Introduction

Executive functions are the cognitive abilities responsible for planning, controlling, and guiding thoughts, feelings, and actions. They are the central executive of the cognitive system, i.e., they transform intentions and purposes into practical actions. People with a greater development of executive functions are more likely to achieve their goals, as they can plan their tasks adequately. There is ample evidence of the impact of executive functions on various areas of human development, especially academic performance (Diamond 2016). Next, we define the theoretical model on which the Yellow-Red test was built, describe the relationship between children and technology use, and summarize previous contributions regarding the assessment of executive functions at the international level. Subsequently, a general description of the test is presented, allowing a better understanding of the results and reflections derived from the standardization process of the Yellow-Red test.

### 1.1. The Three-Component Model of Executive Functions

There are various models for conceptualizing executive functions; one of the most widely accepted is the one that defines the presence of three basic components of executive functions. These basic components develop interdependently during childhood and early adolescence and serve as the basis for developing higher-order executive functions such as planning and problem-solving.

Inhibitory control includes inhibiting thoughts, actions, or behaviors in the face of competing for internal or external stimuli. It is, therefore, an ability that allows the inhibition of cognitive, emotional, and behavioral factors. The first and second are related to thinking and memory. The third, with behavioral inhibition, for example, is related to gratification delay.

Working memory is the ability to operate with mental representations, whether visual, auditory, or episodic. According to Cowan (2017), it is a set of components of the mind that hold a limited amount of information temporarily available for processing the task at hand. It is an ability of limited capacity, although it progressively expands with age, reaching its maximum capacity around age 12. 

Finally, cognitive flexibility is the ability to provide alternative solutions to the same problem. It is closely related to creativity, and of the three functions, it is the one with the latest appearance (Diamond 2013). 

**Figure 1:**
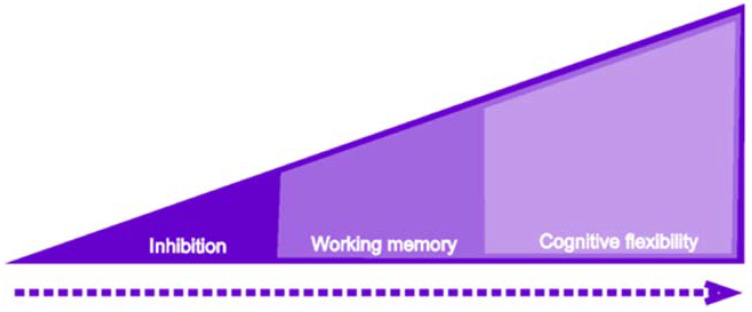
The components that together make up executive functions are shown.

The three main components of EF, as shown in Figure 1, can be understood as successively integrated over time. The first to make its appearance is inhibitory control, followed by working memory, and finally, cognitive flexibility. Although, conceptually, the three components are present throughout development, it is clear that their nature changes from 18 months of age, which is the age at which language begins to be a fundamental part of cognitive development. Language plays a fundamental role in executive functioning since it allows for labeling internal instructions that inhibit behaviors and actions, processing problems, and seeking alternative solutions to unknown problems.

The three components of EF also have important interrelationships, given by their successive integration into development. For example, to solve problems in working memory, it is essential to have active interference control of internal and external stimuli while processing the solution, which is a component of inhibitory control. For this reason, many models of working memory (e.g., Kane and Engle 2003) incorporate interference control as a component of working memory. However, it could properly be considered as a factor of inhibitory control. As Diamond (2013) noted, some authors (e.g., Baddeley and Hitch 1994) incorporated inhibitory and flexibility factors in their working memory model. However, following this author, we kept the three factors separate in the present work, as Miyake et al. (2000) suggested. 

Likewise, cognitive flexibility requires both working memory and inhibitory control to provide the alternative solution being processed (e.g., if the task is to say all the words that begin with a given letter, in working memory, I must simultaneously evaluate that the new term I come up with actually starts with that letter and simultaneously remember and discard repeating the ones I have already said).

An interesting issue regarding the progressive differentiation of executive functions is the unicity versus diversity approach formulated by Friedman and Miyake (2017). Essentially, this approach postulates that executive functions show unicity and diversity, depending on both the analysis techniques used and the developmental level of the samples assessed. We stayed with the latter aspect for the present article, which focuses mainly on children. Friedman and Miyake (2017) reported that, although some studies show a unicity of executive functions at early ages, all studies evidence a differentiation of working memory and flexibility in older children or adults. In other words, at earlier ages, the appropriate mode for understanding executive functions is unity, while at older ages, it is that of diversity. The evidence on this point is mixed, specifically regarding when and which components are part of the first and second factors in school-aged children. This is partly due to the variety of tests used, as the selection of tests according to each component of executive functions is highly heterogeneous, both in their assessment objectives and the way they are assessed (for a comprehensive review on this topic, see Lee et al. 2013).

### 1.2. Use of Technology Tools for Child Assessment

New generations grow up immersed in digital media-rich environments, and technology is integral to their lives from birth (Sweeney and Geer 2008). From a very young age, children are exposed to technological resources, which translates into an early mastery of various digital tools (Mcmanis and Gunnewig 2012). 

The use of technological tools has increased both in the world and in Chile. In the USA, in 2018, 85% of households had an internet connection (United States Census Bureau 2021). In Chile, the statistics are similar, with 87.4% of households reported to have an internet connection in 2017. Likewise, access to technological devices is equally high, with 85.7% of households in which school-age children live have a smart mobile device (Subsecretaría de Telecomunicaciones de Chile 2017). According to Chaudron et al. (2018), who investigated the use of technology in children aged 0–8 years in 21 countries, the use of digital technologies starts earlier and earlier (under two years), and tablets and smartphones are the preferred devices of children, due to their multifunctionality and portability. According to these authors, devices with touch screens are appreciated by children, especially for their ease of use, the possibility of accessing different applications, and their playful aspects.

For these reasons, the need to incorporate technology into educational systems has been raised (Sweeney and Geer 2008), considering its use both for the mediation of teaching and for the assessment of learning. Day et al. (2019) noted that there is a need for technology- and game-based executive function assessment tools that can be used outside of the clinical or academic context, allowing for accurate, ecological, and contingent assessments.

Technology-mediated assessments have several advantages over traditional assessments, as they allow for gamification of the assessment format by incorporating aspects traditionally related to video games or applications. They also allow the standardization of certain technical elements, such as instructions, examples, or forms of response, and the automation of correction processes. On the other hand, it was observed that the use of technological instruments allows the assessment of aspects impossible to assess and apply in pencil and paper instruments, for example, reaction times, presentation of algorithmically programmed items, and the measurement of aspects related to behavior (Germine et al. 2019; Parsey and Schmitter-Edgecombe 2013). 

Parsey and Schmitter-Edgecombe (2013) noted that the widespread access of younger generations has produced a cohort effect, in which children and young people perform better on computer-based assessments than older people with less technological experience. Moreover, the use of technology in assessment contexts generates an increased level of student engagement and motivation, enabling the expression of their full performance potential (Perrotta et al. 2013; Rosas et al. 2015). 

Germine et al. (2019) recommended focusing on four aspects when developing technology-based assessment instruments: (a) designing interfaces that are accessible and appealing to the target age group, (b) developing simple and clear instructions, (c) presenting applied test items that allow the user to interact with the test, which is more effective than reading written instructions, and (d) developing specific norms for technology-based tests rather than adapting norms from pencil-and-paper instruments. 

However, it is important to consider whether technology-mediated assessments correspond to those in traditional formats. In this regard, several meta-analyses involving tests with students from K to 12 have shown no significant differences in the results of the two types of assessment (Kingston 2009; Wang et al. 2008), which contributes to the reliability of this type of instrument.

### 1.3. Description of Instruments and Gold Standards for FE Assessment 

Multiple research fields have approached executive functions, such as neuropsychology, cognitive psychology, education, and, more recently, cognitive neurosciences. Likewise, each of these areas has developed its assessment paradigms, depending on the nature of their studies. 

The first research came from neuropsychology and was based mainly on studying adults with some type of brain injury, thus establishing the relationship between executive functions and the frontal lobe. The works of Luria and his collaborators were paradoxical. They described frontal lobe syndrome in 1964, proposing a series of tasks to evaluate the relationship between neurological disorders and performance in cognitive and motor functions (Canavan et al. 1985). Thus, in 1980, the standardized version of their procedures was published as the so-called Luria-Nebraska battery. In 1981, they presented the first version for children between 8 and 11 years of age (Plaisted et al. 1983). According to Zelazo et al. (2016), interest in assessing executive functions in children only arises when the belief that the limited frontal lobe development during childhood was demystified in the early 1980s. It has been shown that it is just the opposite since it has been demonstrated that the frontal lobe shows a more significant development during childhood. 

From this new interest in the association between executive functions and frontal lobe development in children, children’s versions of instruments used to assess executive functions in adults were developed. An example is the Stroop Interference Test (Stroop 1935), one of the most widely used neuropsychological measures. The Stroop test consists of three consecutive tasks: First, a list of colors expressed in words must be read aloud. Second, one must name a series of colors presented as such in rectangles. Third, a list of colors printed in ink of a color different from that expressed by the word, for example, “yellow” printed in red ink, must be named. According to Homack and Riccio (2004), there is consensus that this last task measures cognitive flexibility and inhibition. The original version proposed by Stroop was interpreted in different ways. One of the most widely used versions is the one developed by Golden (1978), standardized in 2003 for adults and 2002 for children. The children’s version can be applied to children aged from five to fourteen years and only differs from the adult version in scoring norms (Moran and Yeates 2018; Rozenblatt 2018). This is an example of how the original tools were designed for application with adults. Their children’s versions are only later adaptations, not instruments directly created for these age groups, which do not have standardized versions. If they do, they present very poor application norms (Carlson 2005). According to Hughes and Ensor (2011), child adaptation from adult tasks runs the risk of losing critical components of executive functions, for example, oversimplifying them or not considering other cognitive aspects that develop in parallel or later, such as the use of language, specifically vocabulary.

Since 2000, research in psychology and neuroscience has grown exponentially, generating several instruments consisting of individual behavioral tasks based on performance (for more details, see Carlson (2005) and Garon et al. (2008)), which have become more accurate thanks to their technological versions applied on PCs and, later, on tablets. One of the most widely used tests is the Hearts and Flowers test, which corresponds to a version of “Dots” originally developed by Davidson et al. (2006). This test consists of three consecutive tasks; in the first block, the person must press a key on the same side on which a heart appears (congruent block); in the second task, he/she must press a key on the opposite side of which a flower appears (incongruent block). Finally, there is a mixed block in which hearts and flowers appear randomly. The individual must follow two rules simultaneously, depending on the stimulus that appears, forming a mixed block. Despite its wide use, Hearts and Flowers does not have norms, validity, or reliability studies (Camerota et al. 2020). 

In 1996, Zelazo et al. (1996) presented the first version of the Dimensional Change Card Sort (DCCS). in which the child is asked to sort a series of drawings, first according to their shape (put a card with a rabbit on top of another rabbit card, regardless of its color) and then according to their color (put a card with a red figure on top of another red card, regardless of its shape). The child must sort 48 cards according to the instruction of the evaluator, who randomly says “shape” or “color.” The DCCS is now part of a free, validated, norm-referenced battery for the North American population aged 2.5 to 85 (Zelazo et al. 2013). This is a digital version, whose only disadvantage is that it is only available for IOS devices. The previous tests are traditionally laboratory-based but more ecological; behavioral measures are generally related to cognitive and educational psychology. It is in these contexts where tests that have not been standardized but are widely used are also used, such as Simon says (Strommen 1973), based on the traditional children’s game, or Head Shoulders Knees and Toes (Cameron Ponitz et al. 2008), in which the child is progressively asked to touch parts of his body in alternating order.

Other instruments that can be used for the assessment of executive functions are the ENFEN (Portellano et al. 2011), which assesses the global maturational development of children between 6 and 12 years of age with the main focus on executive functions. This test presents norms for the Spanish population with an individual application format with attractive tasks for students, does not directly consider a play format, and does not use digital support. On the other hand, an alternative is the Psychology Experiment Building Language (PEBL) platform, which allows the free programming of digital tests. This platform has some traditional tests pre-designed on the platform, focusing on evaluating executive functions. Among the tests that can be selected is a version of Berg’s Card Sorting Test, similar to the Wisconsin test, Corsi’s block test, and an implementation of Eriksen’s Flanker task. However, although these tools are digital and free of charge, prior knowledge is required to select the tests to be applied, and they do not present information regarding the norms for each population.

On the other hand, tests that assess more general skills are used in educational contexts, which sometimes include the assessment of executive functions or some of their components. This is the case for tests such as the Woodcock-Muñoz battery (Muñoz-Sandoval et al. 2005) and the WISC-V test (Rosas et al. 2022), which include specific components related to the assessment of executive functions. Finally, and especially in school contexts, some scales assess executive functions indirectly, in different contexts, and through the appreciation of actors close to the children, such as teachers or relatives. Among the most widely used are the Behavior Rating Inventory of Executive Function (BRIEF, Gioia et al. 2000), the Behavior Assessment System for Children, now in its third edition (BASC, Reynolds and Kamphaus 2015), and the Conners test (Conners 2008). 

However, the assessment systems mentioned above present certain limitations because, on the one hand, the tests used in the research area assess executive functions in a general way without detailing aspects related to their components. On the other hand, the assessment of executive functions in school contexts only considers executive functions as a minor aspect of more general skills, such as cognitive ability. Moreover, the scales that focus on the appreciation of third parties tend to mark a tendency towards the less cognitive aspects of executive functions, generating a biased view of their development. On the other hand, there are doubts about the validity of these instruments, which are discussed in the next section. 

### 1.4. Discussion of the Importance of Direct EF Assessment over Indirect Ones

Executive functions are important for children’s behavior and learning, but what method is best for assessing these abilities? Much research shows low correlations between the results of direct and indirect assessments of executive functions. In a review of 20 studies reporting correlations between the two types of measures, Toplak et al. (2013) found that only 24% of all reported correlations were statistically significant and that the median correlations were only r = 0.19 (equating to only 3.6% common variance). It should be noted that this result cannot be attributed to the lack of reliability or validity of both methods since both indirect and direct scales showed quite good psychometric properties. So, how can two types of assessment that are supposed to measure the same thing have such low correlations? A recent study sought to answer this question. Even though both measurement forms can show good predictive abilities for academic performance (Gerst et al. 2015), the evidence seems to indicate that direct cognitive tests are more efficient and robust than indirect assessments for measuring executive functioning. The study by Soto et al. (2020), conducted with 136 children, clearly showed how executive function assessments made by teachers adequately predicted students’ academic assessments (also made by teachers) but failed to predict academic performance. Tests of executive functions instead predict academic performance very well and predict academic ratings even better than indirect assessments of executive functions.

This study is of particular relevance since, to date, it is the only published research with two independent and two dependent variables and, in both cases, with direct and indirect methods. Moreover, this makes it possible to elucidate more precisely what both techniques measure; the academic assessments seem to better measure better school adjustment according to teachers, while the direct ones are a better measure of school adjustment and academic achievement. Thus, it would appear that direct measures are more accurate and would be a better indicator of executive functioning than indirect measures.

### 1.5. Brief Description of the Yellow-Red Battery

The Yellow-Red battery consists of six tests focused on the general assessment of executive functions and the specific assessment of their different components. The assessment system is based on technological support (Tablet) and is within the paradigm of invisible assessment through play (Rosas et al. 2015). The test was designed to be applied to children aged 6 to 11 years and has a total application time ranging from 15 to 30 min.

#### 1.5.1. Cat-Dog

**Figure 2:**
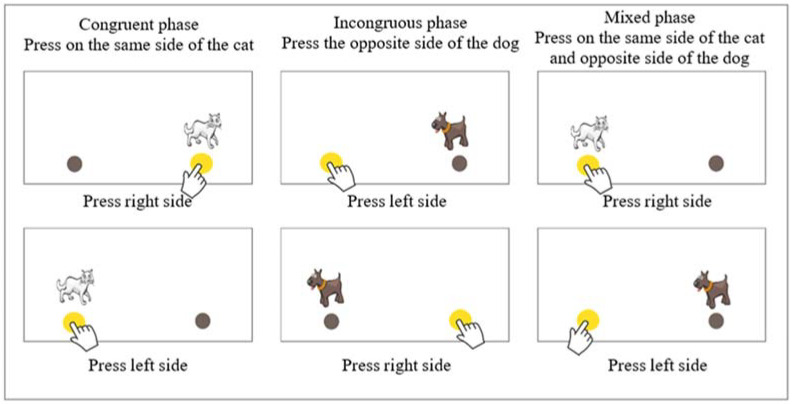
Description of the phases of the Cat-Dog test.

The first test, called Cat-Dog, is an adaptation of Diamond’s Hearts and Flowers test (Diamond 2013). This test theoretically measures the three components of executive functions in its three phases. In the first congruent phase, participants must touch the same side of the screen where a stimulus (cat) appears. The second phase is incongruent: participants must press the opposite side of the screen to where the stimulus appears (dog). In each of the first two phases, 12 cats or dogs appear. In the third phase, congruent and incongruent stimuli (cats and dogs) appear randomly 33 times. In all phases, the stimuli are displayed for 1 s with an interval of 500 milliseconds. Points are only awarded for the results obtained in the third phase. One point is assigned for each correct response, and 0 points are assigned for omissions and incorrect or anticipatory responses, i.e., those executed by the participant before 200 milliseconds elapse. As seen below, this test theoretically evaluates the flexibility component of executive functions (Figure 2).

#### 1.5.2. Arrows

**Figure 3:**
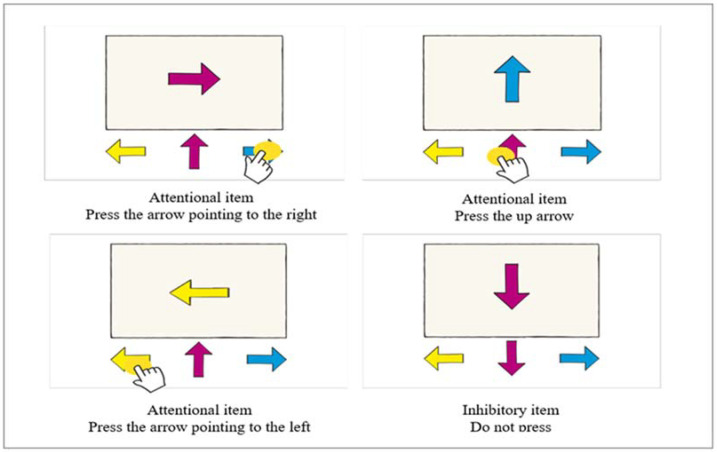
Description of the items of Arrows test.

This test evaluates cognitive inhibition and attention; a “model” arrow and three arrows that function as response alternatives appear on the screen. The arrows point to the right, to the left, up, or down. In the first three cases, children must press the arrow pointing in the same direction as the model. However, the participants should not press anything when the arrow points downward. This test has 36 items, 8 of which correspond to inhibition tasks. The first 15 items are displayed for 2 s, with 500-millisecond intervals, while the following 21 items are presented for 1 s, with 500-millisecond intervals. One point is awarded for each correct response, and 0 points for incorrect or anticipatory response (response with a reaction time less than 200 milliseconds) (Figure 3).

#### 1.5.3. Flies

The Flies test assesses behavioral inhibition using a delay of gratification. A screen is presented with flies flying in different directions, and the participant is asked to smash as many as possible. The flies make a buzzing sound as they fly, and when smashed, they make a sound that the children find very amusing and rewarding. When a green light is turned on, the participant can continue smashing the flies; however, when the light turns red, the participant should not continue smashing the flies. The traffic light changes color, and the participant must follow the rule. When it is green, you can smash flies; when it is red, you cannot.

**Figure 4:**
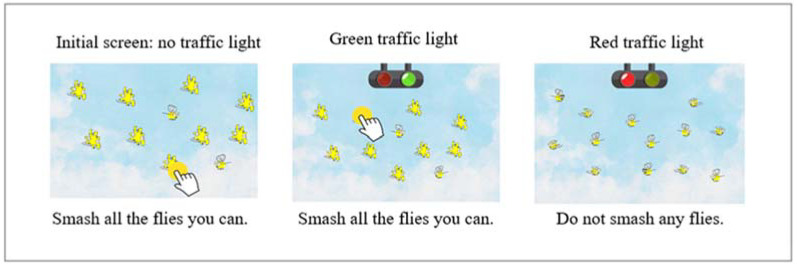
Description of the Flies test.

The test lasts 2 min and is divided into eight different time-lapses where the red or green light appears. Each time-lapse lasts between 3 and 10 s. One point is awarded for each fly smashed. The delayed gratification indicator is the sum of the flies smashed in the green minus those smashed in the red-light time lapses (Figure 4).

#### 1.5.4. Binding

**Figure 5:**
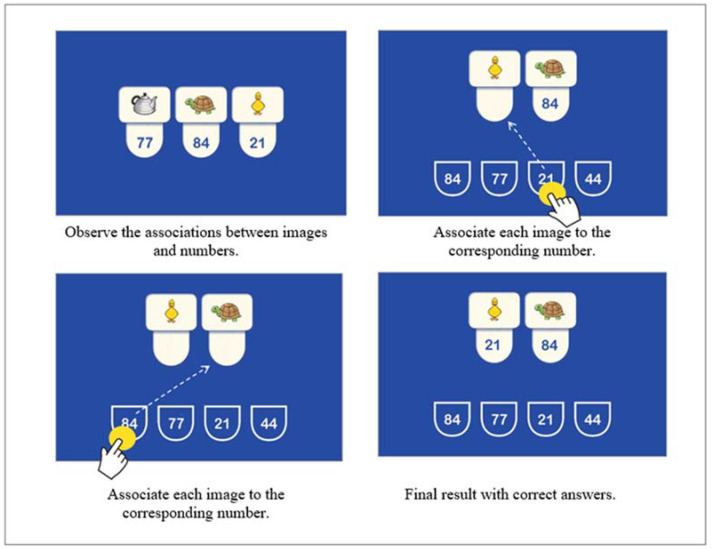
Example of Binding test item.

This test evaluates the development of visuospatial working memory in the form of associated pairs. A series of images related to numbers or geometric figures are presented. Then, some of the stimuli are presented again in isolation, and the participants must establish the associations according to how they were initially presented. The test has 27 items; as the test progresses, more images and numbers are added. In the case of the youngest children (6 to 8 years old), the first five items use geometric figures instead of numbers. From age nine onwards, only items with numbers are presented, and 0 points are assigned for each incorrect answer. An answer in which all pairs are appropriately associated is considered correct; if there is at least one mistake, the item is considered incorrect (Figure 5).

#### 1.5.5. The Farm

**Figure 6:**
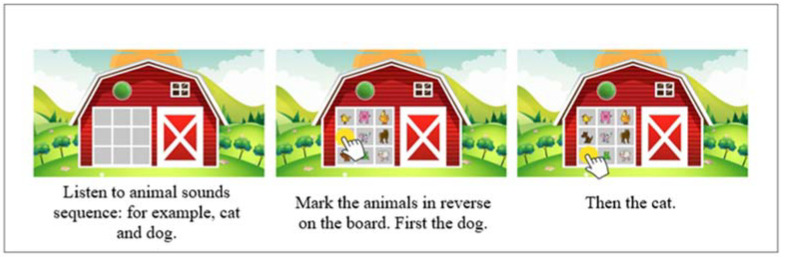
Example of Farm auditory test item.

This test evaluates auditory and visual working memory. The evaluation of auditory working memory is performed by presenting a sequence of animal sounds, after which the participant must select the corresponding animals on a board starting from the last sound heard (Figure 6).

A keyboard is displayed on which some keys light up to assess visual working memory. The participant must press the keys in the reverse order in which they are illuminated.

**Figure 7:**
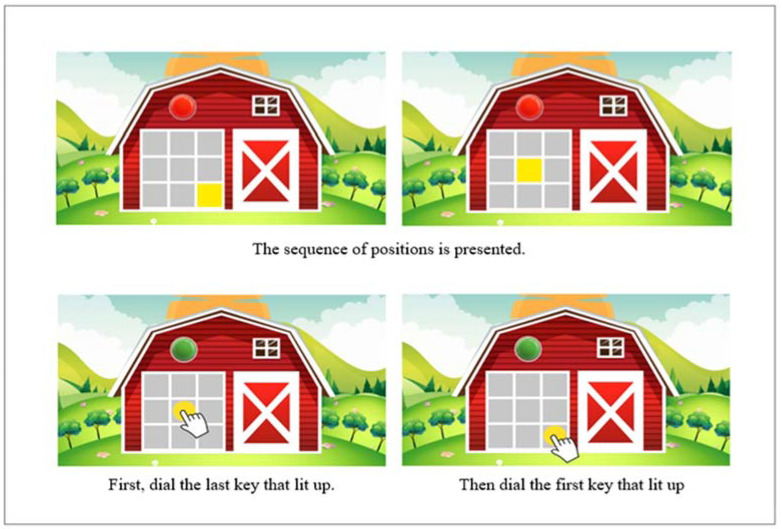
Example of Farm visual test item.

The auditory sequences range from 2 to 8 sounds, and the visual sequences from 2 to 10 visual stimuli. There are 18 auditory items and 18 visual items. One point is assigned for each correct answer. The test is failed when two consecutive errors are made at the same level (the level is determined by the number of sounds to be remembered by the participant) (Figure 7).

#### 1.5.6. Triads

**Figure 8:**
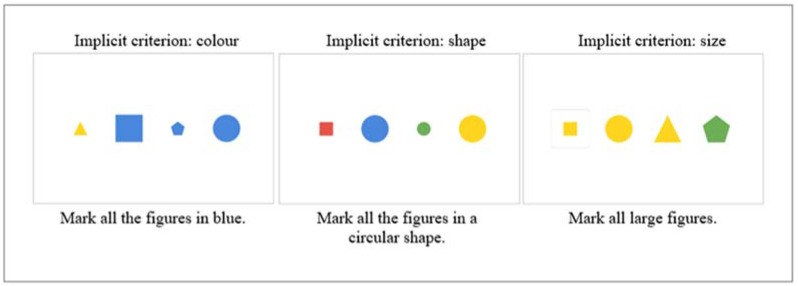
Description of the *Triads* test levels.

The Triads test is oriented to evaluate cognitive flexibility. A series of four geometric figures are presented, three of which have a common characteristic (color, shape, or size). Participants must choose three that have something in common, but the classification criteria are not made explicit. These implicit criteria are color, shape, and size. The criteria change without giving any warning. In total, the test has 21 items, 5 of which correspond to the implicit criterion of color, 5 to the implicit criterion of shape, and 5 to the implicit criterion of size. Six random criterion items follow this. Participants have three chances to get it right; if they fail, they skip to the next category, and those omitted items are considered. Each failed attempt is considered an attentional error, but if it fails all three attempts, it is considered a perseverative error. One point is obtained for each correct answer in the first attempt; in the second attempt, 0.6 points, and in the third attempt, 0.3 points. One point is deducted for each attentional error and 2 points for each perseverative error. There is no time limit for the permanence of the items. This test is suspended after three incorrect answers (Figure 8).

In accordance with the evidence reviewed and the presentation of the instrument developed and standardized to assess executive functions in school-aged children, this study has the following objectives: firstly, at a general level, to demonstrate the importance of having direct standardized measures of executive functions that can be used with children of a wide age range; secondly, to obtain the psychometric properties of the Yellow-Red Test. Finally, we sought to clarify the factor structure of executive functions in Chilean children between 6 and 12 years of age.

## 2. Methodology

The Chilean standardization of Yellow-Red had a meticulous design to have enough information to validate the test with the gold standards for each of the components of executive functions: inhibition, working memory, and flexibility. These gold standards provide valuable information regarding the evidence of validity with other variables and the validity of an instrument developed under the stealth assessment paradigm (Rosas et al. 2015).

### 2.1. Instruments

A table summarizing the correspondence between the Yellow-Red subtests and the respective gold standards applied in the study is presented in Table 1.

#### 2.1.1. Hearts and Flowers

This test assesses executive functions in general and specifically, according to the assessment block. For the present research, only the third phase was used, which assesses cognitive flexibility (A. Diamond, personal communication, April 2018). The Inquisit Web 6 platform (Millisecond Software LLC 2021), which allows offline tablet assessment, was used. Specifically, the Chilean Spanish language version was programmed as described by the original authors of the instrument (Borchert 2021; Diamond et al. 2007). Participants see a set of items in which a heart or a flower appears on the right or left side of a fixation cross. If the person sees a heart, he or she must press a “button” on the tablet on the same side as the heart, which is called a congruent item. On the other hand, if the person sees a flower to the right or left of the fixation cross, he/she must press the button on the opposite side from where the flower appears, which is called an incongruent item. The test consists of three blocks: the first is congruent, in which 20 items (hearts only) are presented, with ten random appearances on each side of the fixation cross. According to Diamond et al. (2007), the congruent block evaluates working memory. The incongruent block also has 20 items, this time only with flowers, with ten flowers on the left and ten flowers on the right of the fixation cross. According to the authors, this block evaluates working memory in addition to inhibitory control. Congruent and incongruent blocks have a maximum response time of 5000 ms. The last block is mixed, and in it, participants must respond to 20 congruent and incongruent items that appear randomly, which assesses working memory, inhibitory control, and cognitive flexibility. The maximum response time in this block is 6000 ms. Each block has three practice items, for which automatic feedback is given to the participant, and failure is a criterion for suspension from the test. For the present study, and due to the differences in presentation times that facilitate a response in the Hearts and Flowers test, compared to Cat-Dog, those correct responses selected 1000 ms after stimulus presentation were scored with 0.5 points (equivalent to half of the correct response). In addition, responses selected before 200 ms scored 0 points.

#### 2.1.2. Flankers

**Figure 9:**
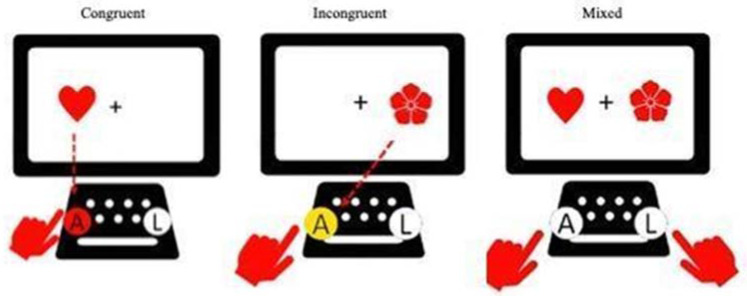
Description of the Hearts and Flowers test phases.

This test evaluates attention and cognitive inhibition. The Inquisit Web 6 platform (Millisecond Software LLC 2021) was used. The test corresponds to the Chilean Spanish version and follows the procedure described by the authors of the instrument (Borchert 2021; Rueda et al. 2004). The test consists of presenting an image of five fish lined up. The participant must pay attention to the fish in the center, and if the fish in the center looks to the right, the participant must press the button on the right. However, if the fish in the center faces left, the participant must press the key on the left. The other fishes in the row can look in the same direction as the fish in the center (congruent item) or in the opposite direction to the fish in the center (incongruent item) (see Figure 9). The platform presents two practice blocks, 12 items in which only four fishes are presented (6 looking left and six looking right), and 12 events with five fishes (three compatible events looking left, three compatible events looking right, three incompatible events looking left, and three incompatible events looking right). The maximum response time per item is 3000 ms. For the present study, and due to differences in presentation times that facilitate responding in the Flankers test compared to Yellow-Red tests that assess inhibition, those correct responses selected after 1500 ms following stimulus presentation were scored with 0.5 points (equivalent to half of the correct response). In addition, responses selected before 200 ms were scored with 0 points.

#### 2.1.3. Modified Card Sort Test

**Figure 10:**
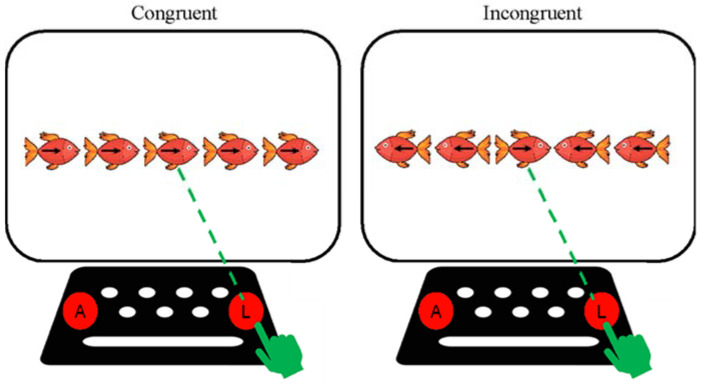
Description of test items *3.

**Figure 11:**
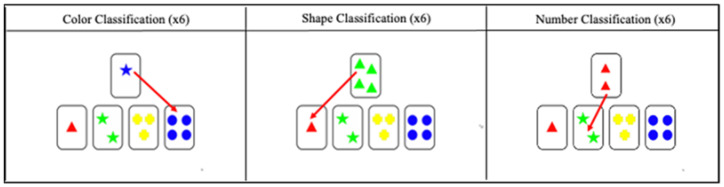
Description of classification categories of the Modified Card Sort Test.

This test assesses cognitive flexibility in the face of changes in the rules of the task. It is a child version of the Wisconsin Card Sorting Test, in which fewer items are considered than in the original version, and only unambiguous cards are presented for sorting. What is essential is the ability to search for a new sorting category when the rule changes implicitly. The Inquisit Web 6 platform (Millisecond Software LLC 2021) was used. The test corresponds to the Chilean Spanish version and follows the procedure described by the authors of the instrument (Borchert 2021; Nelson 1976). The test consists of the subject having to classify one letter per item according to the similarity in a category with one of the four letters displayed below. Two blocks of 24 items are presented, and each of the 24 cards presented in each block has a maximum of one characteristic in common with the four response cards; thus, there are no ambiguous items. The cards in each block are presented randomly and without repetition. For each block, the same order of sorting criteria is followed: color, shape, quantity, repeating the pattern consecutively (see Figure 10). The rule changes automatically after six consecutive correct answers in each category. The score corresponds to the number of correct answers (Figure 11).

#### 2.1.4. Digit Span

This test corresponds to a subtest of the Wechsler Intelligence Scale for Children, 5a edition (WISC-V), in its standardized version for the Chilean population (Rosas et al. 2022). This subtest corresponds to one of the two tests that make up the working memory index (Rosas and Pizarro 2018). It comprises three tasks: (1) Digits in Direct Order: a sequence of numbers is read to the participant, which he or she must repeat in the same order. (2) Digits in Reverse Order: the second sequence of numbers is read to the participant, which the person must repeat in reverse order. (3) Sequenced Digits: in the third sequence of digits, the participant must repeat them in ascending order. Each task includes two practice attempts to ensure the understanding of the task. Each of the three tasks has nine items, each containing two attempts. As the items progress, they have more items to remember; for example, item 1 has numbers, while item 9 has ten numbers in the Digits in Direct Order task. The test has a suspension criterion, which is applied when the child makes a mistake in two attempts at the same item. The total score corresponds to the sum per task of each correct attempt (one point) answered by the subject.

#### 2.1.5. Verbal Fluency Test

Researchers decided to develop a task to assess verbal cognitive flexibility. The test consisted of naming as many items that met specific characteristics as possible in 60 s. The instruction given to the children was the following: “Please name as many things that you like as fast as you can”. The answers were recorded to be scored later, considering as a score the sum of words expressed within the time limit. This test is not part of the Yellow-Red battery; it was developed to have a second test of verbal flexibility to contribute to the factorial structure of the model.

#### 2.1.6. School Adaptation Index

This index corresponds to a composite score between the TRF and the average grades obtained by the student the year prior to the evaluation. The index is expressed in percentiles, in which each measure weighs 50%.

**Teacher Report Form 6–18, Spanish version (TRF):** To obtain information on school adjustment, four questions were used that are not part of the questionnaire itself but of a section of contextual questions on general aspects of the adjustment observed by the educator, such as the degree to which the educator perceives that the student makes an effort, if the student seems happy, if he/she behaves appropriately, etc. The questions were answered in Likert format, with zero points indicating “Much less than the average of their peers,” while a score of seven indicated “Much less than the average of their peers” (Achenbach and Rescorla 2001). Thus, the maximum possible raw score was 28 points. This score was transformed into a percentile so that a score of 28 points was equivalent to 100% TRF-Adaptation.

**Academic Performance:** The second measure of school adjustment corresponds to the grade point average obtained by the student in 2020. This average ranged from 0 to 7 and was reported by the students’ schools. The average obtained was transformed to a percentile so that grade seven corresponded to 100% academic performance.

### 2.2. Sample

The Yellow-Red standardization process was approved by the Scientific Ethical Committee of Social Sciences, Arts, and Humanities of the Pontificia Universidad Católica de Chile. Sampling focused on subjects having sufficient variability in the following characteristics: SES, gender, age, and school adjustment, for which the individual school adjustment index described in the previous section was used. Table 2 shows the sample distribution according to SES, gender, and level of education of each student.

The sample consisted of 245 participants (122 girls); 110 belonged to the low SES and 135 to the high SES. The socioeconomic categorization proposed by the Quality Agency of the schools attended by the students was considered to define their SES. This categorization considers the parents’ educational level, the family’s average monthly income, and the vulnerability index. This index was calculated based on the percentage of students in the school who are in extreme poverty or at risk of school failure. The first three indicators were obtained through a survey answered by the families of the children who took the SIMCE test (a national standardized test to assess Math and Language). The last one was obtained from the Junta Nacional de Auxilio Escolar y Becas (JUNAEB) data.

The students came from schools in Santiago, Chile, in grades from kindergarten to 6th grade.

All students took all the tests. Only two children did not participate in the Flies subtest. These missing cases are due to one child with suspected color blindness (necessary for the test) and one child not responding.

The principal of each school agreed to participate in the study and gave the authorization to contact students’ families. All participants were authorized by their parents or legal guardians through a letter of informed consent; they also went through the informed consent process.

### 2.3. Procedure

Students were assessed at home or at their educational establishments during school hours in a room provided by each school to carry out the procedure. The evaluations were carried out at the end of the current school year (second semester 2021). The assessment was conducted in one 60-min session for students in grades two through six and two 30-min sessions for those in kindergarten and first grade. A trained evaluator administered all tests individually in oral or digital format (Tablet format).

Traditional format tests were administered first, i.e., oral question-answer tests. These were the subtests of the WISC-V. In this case, the evaluators recorded the answers on the Tablet, and after the evaluation, they scored the tests. Subsequently, the tests were applied in digital format; first were the gold standard tests, Hearts and Flowers, followed by the Flanker test and the Card Sort Task, which were presented randomly, according to the definition in the programming of the application. Then, the Yellow-Red battery tests were applied in the following order: Cat-dog, Triads, Arrows, Binding, Farm (auditory and visual), and Flies. Finally, the verbal fluency test was applied.

Regarding missing data, the analyses were carried out considering the two students mentioned above as missing cases and using the listwise method for the multivariate analyses.

The data analysis was performed with SPSS 27 for all analyses except the confirmatory factor analysis performed with Mplus 8. The analysis plan was structured as follows: to determine internal consistency, a reliability test was performed. To assess the validity of Yellow-Red, the progression of scores according to age was evaluated, using the course attended by the students as a proxy for this variable. The difference in scores for each test between kindergarten, third, and fifth grade was reported. To obtain evidence of convergent and discriminant validity of the Yellow-Red test, an analysis of correlations with reference variables (gold standards) and with a variable that theoretically did not correlate with the instrument was carried out. To check the structure of the test, three confirmatory factor analyses were carried out: for the full sample, for younger students, and for older students.

## 3. Results

### 3.1. Evidence of Reliability

As can be seen in Table 3, the evidence of test reliability is excellent, both in internal consistency indicators and in bipartition indicators (for the Flies test, an internal consistency indicator could not be calculated). In five of six tests, the coefficients exceeded 0.8, which is considered very good. In the Cat-Dog test, the value was above 0.9, which is considered excellent.

### 3.2. Evidence of Validity

#### 3.2.1. Progression of Executive Functions with Age

**Figure 12:**
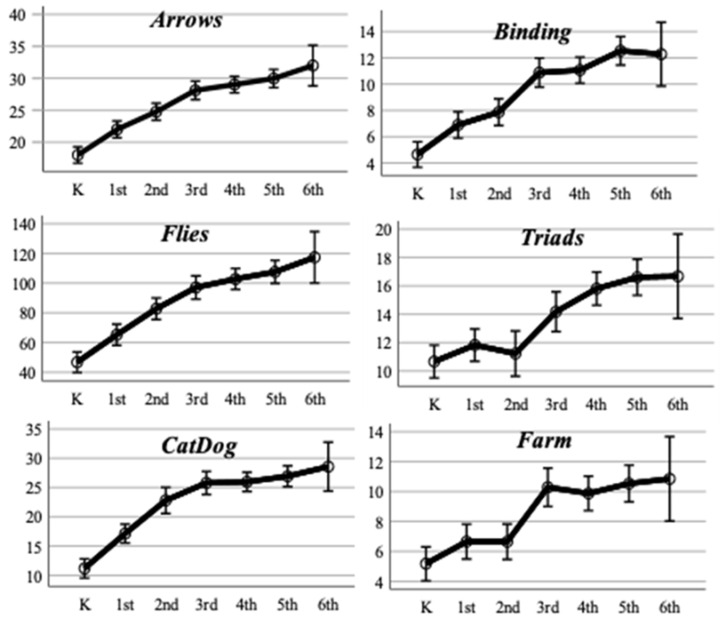
Age progression of estimated marginal means by subtest.

The progression of the results according to age showed clear evidence of the tests’ validity. As shown in Figure 12, all the tests, except for Farm, showed an evident progression concerning age. Although, in all of them, a flattening of the curves was observed towards older ages, which may indicate that the test decreased its discriminative capacity as the age of the children increases. Regarding Farm, it reached its highest value in third grade and practically did not increase in the following grades (Figure 12).

A one-way ANOVA was performed to test the progression of scores according to age. The test was conducted considering kindergarten, second, and fifth grade, since the sample size decreased in sixth grade. As can be seen in Table 4, all tests showed a statistically significant increase in scores according to age. Additionally, the effect sizes were all above 0.14, which is considered large. The post hoc Tukey analysis indicated that the difference of 1.47 points (95% CI [−3.28, 0.34] *p* = 0.136) on average between kindergarten and second grade on the Farm test was the only comparison that was not statistically significant.

#### 3.2.2. Evidence of Convergent and Discriminant Validity

Table 5 presents the correlations of the YR tests with their corresponding gold standards and with a test that theoretically should not correlate significantly with executive functions, e.g., the mental health variable as perceived by teachers on the TRF scale. This variable corresponds to the response to the question to what degree is (the student) happy and content? As can be seen in Table 5, all the correlations observed were significant at 1%, with values ranging from 0.42 (flexibility) to 0.73 (behavioral inhibition). Likewise, all the correlations with the discriminant test were close to zero and insignificant.

#### 3.2.3. Evidence of Factorial Validity

The factorial structure of executive functions during childhood and early adolescent development goes from being a unitary construct to being progressively differentiated into two factors. Later in adolescence and adulthood, it shows a structure in which three differentiated factors emerge but maintain a certain level of correlation between them (Lehto et al. 2003; Shing et al. 2010; Willoughby et al. 2012). In the present study, we sought to test whether the Yellow-Red subtests also reflected the increasing diversification of the components of executive functions with age. For this, a series of CFAs were carried out, which allowed for interpreting the relationship between observed variables (in this case, the results in each of the Yellow-Red tests) with latent variables or the components of the executive functions, and the relationship existing between the latent variables. The three latent variables defined are: inhibition (I), for which the observed variables corresponded to the results of the Arrows (ARR) and Flies (FLI) subtests. The latent variable working memory (WM) was composed of the observed variables Binding (BIN) and Farm (FAR), while the latent variable cognitive flexibility (CF) was composed of Triads (TRI) and Cat-Dog (CAT). The models tested ranged from most differentiated to least differentiated. Thus, the first model evaluated a structure of three latent factors. The second model was composed of two factors, in which two components were combined into one latent variable and a third factor was left with only one latent variable. This model had three versions, in order to test all possible combinations between components. Finally, the simplest model was the one with a single latent variable, which grouped all the components of executive functions (see Table 6).

To test the increasing differentiation in the components of executive functions, the analysis was carried out in three groups; one considered the entire sample, i.e., students from kindergarten to sixth grade. The second group considered the youngest children in the study, kindergarten and first grade students. Finally, the analysis was conducted with the older children, students from second to sixth grade. The CFA was performed using a robust maximum likelihood estimator (MLM), which allows the analysis to be performed with variables that present distributions with a certain level of abnormality. For each group analyzed, a table is presented with the five models and their respective adjustment statistics. The model with the best fit to the data was highlighted, which was then represented graphically, indicating the parameters for each of the variables.

Each of these models was tested under three conditions: the first condition considered the whole sample, the second condition considered only the youngest children in the sample (kindergarten and first grade), and the third condition included only the oldest students (second to sixth grade).

**Confirmatory factor analysis: Kindergarten to sixth grade**

**Figure 13:**
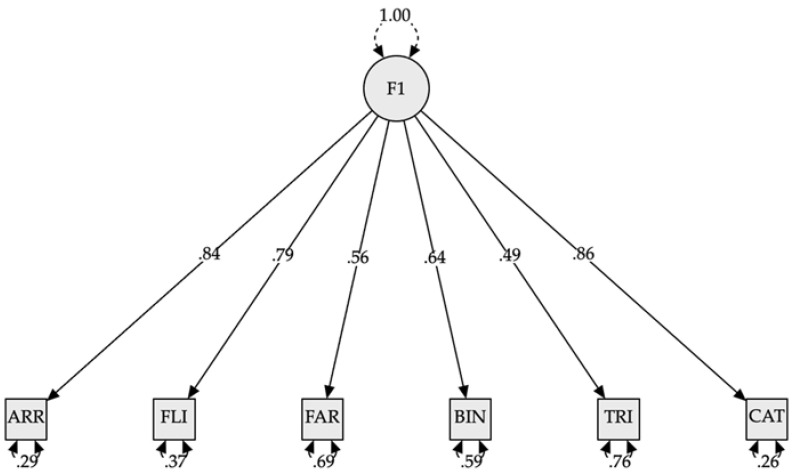
Model plot confirmatory factor analysis (kindergarten to sixth grade). *Note.* F1 = Model 3 (I + WM + CF). ARR = subtest Arrow. FLI = subtest Flies. FAR = subtest Farm. BIN = subtest Binding. TRI = subtest Triads. CAT = subtest Cat-Dog. All modeled correlations and path coefficients are statistically significant (*p* < 0.001).

The results shown in Table 7 indicate that, for the whole sample, the model that best fit the data corresponded to model 3 (see Figure 13), which considered a single factor that included the three components of executive functions. Models 1, 2.A, and 2.B presented latent factors with correlations greater than 1, which prevented an accurate estimation of their degree of fit. In this case, it was recommended to collapse the factors that presented problems. Models 2C and 3 presented statistically significant χ^2^ values and *p*-values, indicating a low level of fit. However, this could be due to the small number of participants, as the χ^2^ statistic is very sensitive to the N of the sample (Byrne 2011). When analyzing the rest of the goodness-of-fit indicators for models 2C (CFI = 0.974; RMSEA = 0.095; SRMR = 0.038) and 3 (CFI = 0.974; RMSEA = 0.089; SRMR = 0.38), we saw that both models presented adequate values for CFI (greater than 0.95) and SRMR (lower than 0.08), but presented difficulties concerning RMSEA (values higher than 0.06 were observed). However, model 3 presented a value closer to what was expected; thus, it was considered the most adequate model.

**Confirmatory factor analysis: Kindergarten and first grade**

**Figure 14:**
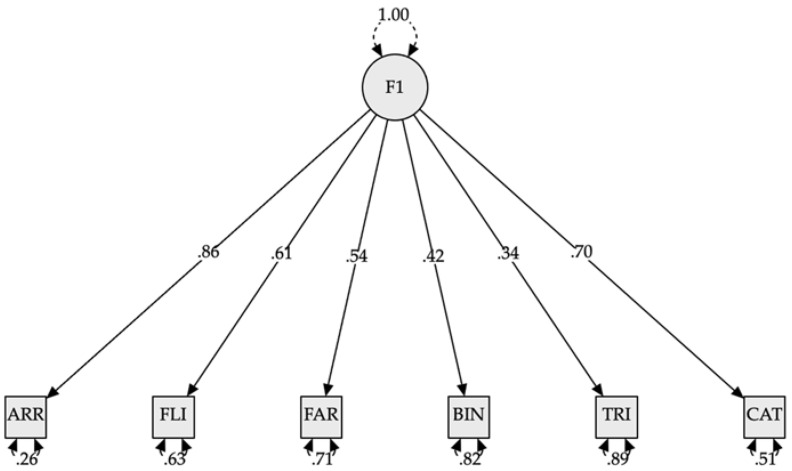
Model plot confirmatory factor analysis (kindergarten and first grade). *Note.* F1 = Model 3 (I + WM + CF). ARR = subtest Arrows. FLI = subtest Flies. FAR = subtest Farm. BIN = subtest Binding. TRI = subtest Triads. CAT = subtest Cat-Dog. All modeled correlations and path coefficients are statistically significant (*p* < 0.001).

As we can see in Table 8, the confirmatory factor analysis results for kindergarten and grade 1 children indicated that the best-fitting model was model 3 (see Figure 14), which indicates the existence of a single latent factor grouping the observed variables. The CFI value of 0.92 indicated an adequate fit. However, RMSEA (greater than 0.10) and SRMR (greater than 0.065) indicated a low model fit. However, this could be due to the small sample size. Models 1 and 2A, 2B, and 2C were discarded because the latent variable covariance matrix (PSI) was not positive definite.

**Confirmatory factor analysis: Second to sixth grade**

**Figure 15:**
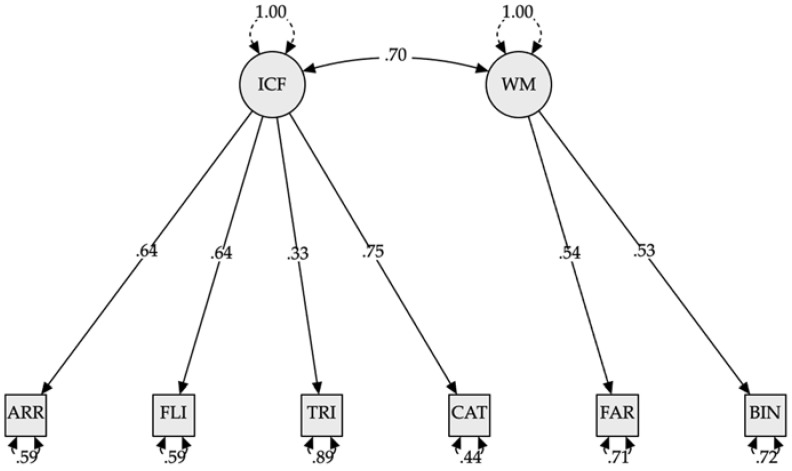
Model plot confirmatory factor analysis (second to sixth grade). *Note*. ICF = Inhibition plus cognitive flexibility, WM = working memory, ARR = Arrows, FI = Flies, TRI = Triads, CAT = Cat-Dog, FAR = Farm, BIN = Binding. All modeled correlations and path coefficients are statistically significant (*p* < 0.001).

The best fitting model for students in grades 2–6 was model 2C (CFI = 0.926; RMSEA = 0.095; SRMR = 0.52). Models 1, 2A, and 2B were unacceptable, as the latent variables had correlations greater than 1. Model 3 was discarded as it had a lower fit than model 2C (see Table 9). Model 2-C is represented in Figure 15.

## 4. Discussion

This article described the Yellow-Red test’s standardization process for assessing executive functions in children aged 6 to 11. The development of this test responded to the need for instruments that allow valid and reliable measurement of executive functions due to the high impact they have on academic performance, as well as on work adaptation and emotional stability in adulthood (Diamond 2016).

The need for instruments designed specifically for child assessment, which also have solid validity and reliability indicators, was highlighted. The Yellow-Red test adequately responded to this demand, being, to our knowledge, the only instrument to measure executive functions playfully, based on a Tablet format, which independently assesses the three basic components of executive functions postulated by Miyake et al. (2000) and has psychometric evidence that supports its reliability and validity.

Yellow-red is a test designed specifically for assessing executive functions in children, and its development considered the four aspects raised by Germine et al. (2019). The Yellow-Red test has several advantages over other assessment instruments. The test is attractive to users because, on the one hand, the use of a digital medium that can be manipulated directly by children takes into account the high technological proficiency demonstrated by students belonging to generations of digital natives (Mcmanis and Gunnewig 2012; Sweeney and Geer 2008). On the other hand, it incorporates playful dynamics, which has been shown to positively impact children’s motivation and engagement (Perrotta et al. 2013). On the other hand, using playful tests with digital support increases the chances of accuracy in the assessment results, especially in the case of children with learning difficulties (Rosas et al. 2015). Finally, as there is no evidence that the digital format interferes negatively with the cognitive assessment process of children (Kingston 2009; Wang et al. 2008), the incorporation of technology and gamification, such as the Yellow-Red battery, is considered relevant.

Although there are other instruments designed to assess the various components of executive functions, both independently and in general, many of these do not have information regarding their psychometric properties, which significantly reduces their reliability. Examples of this are Espy’s Shape School test (Espy 1997), which assesses flexibility and inhibition in preschoolers; the Dimensional Change Card Sorting Test (DCCS) (Zelazo et al. 1996), which assesses flexibility; and the Hearts and Flowers test, which theoretically assesses the three components, initially called Dots (Davidson et al. 2006; Diamond et al. 2007).

On the other hand, they are standardized instruments with evidence of reliability and validity. However, they are oriented only to one of the components of executive functions, or they frame their evaluation as a part of a more general cognitive function. This is the case for the WISC-V Digit Span subtest, which assesses working memory in isolation based on the application of three subtests (digit span forward, backward, and sequencing), of which strictly only the last two assess working memory (the first assesses short term memory); for the Woodcock-Muñoz number reversal and auditory working memory subtests, which are oriented to the assessment of working memory; and for the concept formation subtest, related to cognitive flexibility (Schrank et al. 2005). Thus, although we have standardized instruments, their design was not focused on evaluating executive functions and even less on the specific assessment of their components. Still, they are oriented to general cognitive skills or school performance.

However, other instruments have several characteristics attributed to the Yellow-Red battery. An example of this is the battery developed by Zelazo et al. (2013), which is also game-based and presented in a Tablet format, and it proposes the evaluation of the three basic components of executive functions; a clear differentiation of the components cannot be made because the total assessment is based only on the use of two tests (DCCS and Flankers), which could generate a contamination of the tasks and therefore difficulty in isolating the performance in each of its components. On the other hand, Yellow-Red has at least one test to evaluate each component, which strengthens the possibilities of differentiation.

One of the main pieces of evidence of the validity of the Yellow-Red test is the excellent correlations obtained with instruments considered gold standards for each component. The convergent and discriminant validity analysis showed conclusive results regarding the quality of each subtest to assess, respectively, the factors of inhibition, working memory, and flexibility.

On the other hand, test results generally show a progression with age. That is, performance improves in direct relation to age. This is in line with the progressive development of executive functions, which has been widely described in the literature (Friedman and Miyake 2017; Miyake et al. 2000; Gathercole 1998). As shown, the onset of executive function development begins during the first months of age and continues into adulthood. The results presented here align with the expected progress in executive function development. One particularly striking result is the performance in the ‘Farm’ test (Figure 12). Here, a clear difference was seen between the results obtained by participants in grades K-2 and grades 3–6. This result may be related to the progressive development of working memory, which increases by one unit of information every two years. Thus, given that there are participants of different ages in each grade, the K–2 group corresponds to participants with an average age of 7.2 years (StdDev = 0.9), and the 2–6 grade group corresponds to participants with an average age of 10.4 years (StdDev = 0.9). A possible explanation for this observed result is that, in the second group (3–6 grades), the development of some skills necessary for a good performance in WM tests has started (Gathercole 1998). In particular, rehearsal and practice-by-repetition strategies, whose development starts at seven years, should be more present in the second group (3rd–6th grade) (Chooi and Logie 2020; Morra 2015).

The confirmatory factor analysis of the six Yellow-Red subtests, plus the one carried out with the gold standard tests, recognizes and reaffirms that Chilean children’s executive function structures progressively differentiate with age. Starting with a common factor at preschool age, as demonstrated by Wiebe et al. (2008) and Willoughby et al. (2012), until around six years of age and differentiating into two factors in middle childhood, from seven to 12 years of age, as found by Shing et al. (2010). One factor linked to inhibition and another related to cognitive flexibility, sharing working memory tasks, can be distinguished. However, as Wiebe (2014) points out, the evidence for two factors is robust, but their composition is not.

Future research should clarify whether, by taking a wider age range, the three factors can be more clearly differentiated. For this, the test should be applied to students up to at least 12 years of age.

## 5. Conclusions

After a rigorous development, adaptation, and standardization process, it was possible to develop a battery for an exhaustive assessment of executive functions, considering global indicators and specific measures for each primary component. All the subtests that compose it have sufficient evidence of validity and reliability. The Yellow-Red executive function assessment battery responds to the needs of the academic, clinical, and educational communities. By having an appropriate assessment instrument, researchers and practitioners can accurately assess children’s executive functions at an early age, which allows for the detection of possible difficulties and the strengthening of skills that could present difficulties.

Future research will explore the longitudinal use of this tool in various contexts and demonstrate the practical impact of a playful, technology-based instrument that allows both the general and isolated assessment of the different components of executive functions. This could imply the consideration of these results in the design of clinical interventions and educational programs, which, considering the broad impact of executive functions in diverse areas of human development, could contribute to the possibilities of correcting difficulties and strengthening those aspects necessary to improve people’s quality of life.

## 6. Limitations

The present study showed strong evidence for the validity and reliability of Yellow-Red. However, the sample size comprised rather small groups, mainly when classified by age and SES. These sample sizes may affect the interpretability and extrapolation of results to more diverse populations. In this sense, complementing the present study with studies conducted in other populations, countries, and cultures would provide information on the instrument’s usability in other cultural contexts.

## Figures and Tables

**Table 1:** Correspondence of Yellow-Red subtests with their gold standards.

General Component	Specific Component	Yellow-Red Subtest	Gold Standard
Inhibition	Cognitive inhibition and attention	Arrows	Flankers
	Behavioral inhibition	Flies	Flankers
Working Memory	Visuospatial working memory	Binding	Digit Span WISC V
	Auditory and visual working memory	Farm	Digit Span WISC V
			Block Design WISC V
Cognitive Flexibility		Triads	Modified Card Sort Test
			Similarities WISC V

**Table 2:** Sample distribution by SES, gender, and grade.

	Low SES				High SES					
	Female		Male		Female		Male		Total	
	N	%	N	%	N	%	N	%	N	%
Kinder	9	3.67	9	3.67	12	4.90	14	5.71	44	17.96
1st Grade	10	4.08	11	4.49	10	4.08	10	4.08	41	16.73
2nd Grade	10	4.08	10	4.08	10	4.08	10	4.08	40	16.33
3rd Grade	3	1.22	7	2.86	12	4.90	12	4.90	34	13.88
4th Grade	9	3.67	12	4.90	12	4.90	9	3.67	42	17.14
5th Grade	10	4.08	7	2.86	10	4.08	10	4.08	37	15.10
6th Grade	2	0.82	1	0.41	3	1.22	1	0.41	7	2.86
Total		21.6		23.2		28.1		26.9	24	100.0

*Note.* SES = Socioeconomic Status.

**Table 3:** Reliability analysis, internal consistency, or bipartition indicator.

Yellow Red	Cronbach’s Alpha	Pearson’s r
Flies		0.82 **
Arrows	0.88	
CatDog	0.91	
Binding	0.86	
Farm	0.82	
Triads	0.86	

Note: Interpretation of Cronbach’s alpha values: α ≥ 0.9, excellent; 0.9 > α ≥ 0.8, good; 0.8 > α ≥ 0.7, acceptable; 0.7 > α ≥ 0.6, questionable; 0.6 > α ≥ 0.5, poor; 0.5 > α, unacceptable. ** = *p* < 0.01.

**Table 4:** ANOVA results.

		*N*	M	SD	SE	*F*(2,118)	η^2^
Arrows	Kinder	44	18.000	4.393	0.662	87.773 ***	0.598
	2nd grade	40	24.775	4.554	0.720		
	5th grade	37	30.108	3.204	0.527		
Binding	Kinder	44	4.659	2.828	0.426	55.644 ***	0.485
	2nd grade	40	7.875	3.750	0.593		
	5th grade	37	12.568	3.516	0.578		
Flies	Kinder	44	46.818	24.819	3.742	68.352 ***	0.541
	2nd grade	40	82.825	24.142	3.817		
	5th grade	35	107.629	20.150	3.406		
Triads	Kinder	44	10.611	2.639	0.398	28.096 ***	0.323
	2nd grade	40	12.810	4.501	0.712		
	5th grade	37	16.600	3.498	0.575		
CatDog	Kinder	44	11.386	5.809	0.876	88.948 ***	0.601
	2nd grade	40	22.063	5.330	0.843		
	5th grade	37	26.500	4.531	0.745		
Farm	Kinder	44	5.182	3.208	0.484	24.789 ***	0.296
	2nd grade	40	6.650	3.393	0.537		
	5th grade	37	10.541	3.884	0.639		

*** *p <* 0.001.

**Table 5:** Correlations between Yellow-Red subtests and their corresponding gold standard and TRF (mental health test, not related to measured skills).

Yellow-Red Subtest	The Gold Standard (Reference)	Correlation	Correlation with TRF
Flies	Flankers	0.59 **	0.02
Arrows	Flankers	0.72 **	0.03
CatDog	Hearts and flowers	0.63 **	0.00
Binding	Digit Span WISC V	0.57 **	0.08
Farm	Digit Span WISC V	0.63 **	0.06
Triads	Modified Card Sort Test	0.42 **	0.12
	Oral Fluency	0.37 **	0.17*

Note WISC V Digits: Mean score obtained in forward, backward, and sequencing digits. Correlation using Pearson’s r: ** = *p* < 0.01.

**Table 6:** Proposed models for confirmatory factor analysis.

Model	Factor 1	Factor 2	Factor 3
Model 1	I	WM	CF
Model 2A	(I + WM)	CF	
Model 2B	I	(WM + CF)	
Model 2C	(I + CF)	WM	
Model 3	(I + WM + CF)		

Note. I = inhibition, WM = working memory, CF = cognitive flexibility.

**Table 7:** Goodness of fit indices for alternative CFA models (kindergarten to sixth grade).

Model	*df*	χ^2^	*p*	CFI	RMSEA	RMSEA 95% CI	SRMR
(1) I-WM-CF ^a^	6	19.545	0.003	0.980	0.096	0.051–0.146	0.033
(2.A) (I + WM)-CF ^a^	8	19.883	0.010	0.982	0.078	0.035–0.122	0.033
(2.B) I-(WM + CF) ^a^	8	25.013	0.002	0.975	0.094	0.054–0.136	0.039
(2.C) (I + CF)-WM	8	25.624	0.001	0.974	0.095	0.055–0.138	0.038
(3) (I + WM + CF)	**9**	**26.308**	**0.002**	**0.974**	**0.089**	**0.051–0.129**	**0.038**

*Note.* ^a^ = The latent variable covariance matrix (PSI) is not positive definite. The model with the best fit is highlighted in bold.

**Table 8:** Goodness of fit indices for alternative CFA models (kindergarten and first grade).

Model	*df*	χ^2^	*p*	CFI	RMSEA	RMSEA 95% CI	SRMR
(1) I-WM-CF ^a^	6	7.772	0.255	0.985	0.059	0.000–0.161	0.041
(2.A) (I + WM)-CF ^a^	8	13.765	0.088	0.950	0.092	0.000–0.172	0.055
(2.B) I-(WM + CF) ^a^	8	18.551	0.018	0.909	0.125	0.049–0.200	0.065
(2.C) (I + CF)-WM ^a^	8	14.601	0.067	0.943	0.099	0.000–0.1770	0.056
**(3) (I + WM + CF)**	**9**	**18.731**	**0.028**	**0.916**	**0.113**	**0.036–0.185**	**0.065**

*Note.* ^a^ = The latent variable covariance matrix (PSI) is not positive definite. The model with the best fit is highlighted in bold.

**Table 9:** Goodness of fit indices for alternative CFA models (second to sixth grade).

Model	*df*	χ^2^	*p*	CFI	RMSEA	RMSEA 95% CI	SRMR
(1) I-WM-CF ^a^	6	16.714	0.010	0.931	0.106	0.047–0.168	0.048
(2.A) (I + WM)-CF ^a^	8	21.337	0.006	0.913	0.103	0.051–0.157	0.053
(2.B) I-(WM + CF) ^a^	8	22.321	0.004	0.907	0.106	0.055–0.160	0.056
(2.C) (I + CF)-WM	**8**	**19.474**	**0.012**	**0.926**	**0.095**	**0.042–0.150**	**0.052**
(3) (I + WM + CF)	9	23.917	0.004	0.903	0.102	0.054–0.153	0.056

*Note.* ^a^ = The latent variable covariance matrix (PSI) is not positive definite. The model with the best fit is highlighted in bold.

## Data Availability

The data presented in this study are openly available in FigShare at https://doi.org/10.6084/m9.figshare.21701687.v1.

## References

[B1-jintelligence-10-00125] Achenbach Thomas M., Rescorla Leslie A. (2001). Manual for the ASEBA School-Age Forms and Profiles.

[B2-jintelligence-10-00125] Baddeley Alan. D., Hitch Graham J. (1994). Developments in the Concept of Working Memory. Neuropsychology.

[B3-jintelligence-10-00125] Borchert Katja (2021). User Manual: Inquisit Hearts and Flowers Task (Chilean Spanish version).

[B4-jintelligence-10-00125] Byrne Barbara M. (2011). Structural Equation Modeling with Mplus: Basic Concepts, Applications, and Programming.

[B5-jintelligence-10-00125] Cameron Ponitz Claire E., McClelland Megan M., Jewkes Abigail M., Connor Carol McDonald, Farris Carrie L., Morrison Frederick J. (2008). Touch your toes! Developing a direct measure of behavioral regulation in early childhood. Early Childhood Research Quarterly.

[B6-jintelligence-10-00125] Camerota Marie, Willoughby Michael T., Magnus Brooke E., Blair Clancy B. (2020). Leveraging item accuracy and reaction time to improve measurement of child executive function ability. Psychological Assessment.

[B7-jintelligence-10-00125] Canavan Anthony, Janota Ivan, Schurrt Paul H. (1985). Luria’s frontal lobe syndrome: Psychological and anatomical considerations. Neurosurgery, and Psychiatry.

[B8-jintelligence-10-00125] Carlson Stephanie M. (2005). Developmentally Sensitive Measures of Executive Function in Preschool Children. Developmental Neuropsychology.

[B9-jintelligence-10-00125] Chaudron Stephane, Di Gioia Rosanna, Gemo Monica (2018). Young Children (0-8) and Digital Technology: A Qualitative Study across Europe.

[B10-jintelligence-10-00125] Chooi Weng-Tink, Logie Robert (2020). Changes in error patterns during n-back training indicate reliance on subvocal rehearsal. Memory & Cognition.

[B11-jintelligence-10-00125] Conners C. Keith (2008). Conners 3.

[B12-jintelligence-10-00125] Cowan Nelson (2017). The many faces of working memory and short-term storage. Psychonomic Bulletin and Review.

[B13-jintelligence-10-00125] Davidson Matthew C., Amso Dima, Anderson Loren Cruess, Diamond Adele (2006). Development of cognitive control and executive functions from 4 to 13 years: Evidence from manipulations of memory, inhibition, and task switching. Neuropsychologia.

[B14-jintelligence-10-00125] Day Jamin, Freiberg Kate, Hayes Alan, Homel Ross (2019). Towards Scalable, Integrative Assessment of Children’s Self-Regulatory Capabilities: New Applications of Digital Technology. Clinical Child and Family Psychology Review.

[B15-jintelligence-10-00125] Diamond Adele (2013). Executive functions. Annual Review of Psychology.

[B16-jintelligence-10-00125] Diamond Adele (2016). Why improving and assessing executive functions early in life is critical. Executive Function in Preschool-Age Children: Integrating Measurement, Neurodevelopment, and Translational Research.

[B17-jintelligence-10-00125] Diamond Adele, Barnett W. Steven, Thomas Jessica, Munro Sarah (2007). Preschool Program Improves Cognitive Control. Science.

[B18-jintelligence-10-00125] Espy Kimberly A. (1997). The shape school: Assessing executive function in preschool children. Developmental Neuropsychology.

[B19-jintelligence-10-00125] Friedman Naomi. P., Miyake Akira (2017). Unity and diversity of executive functions: Individual differences as a window on cognitive structure. Cortex.

[B20-jintelligence-10-00125] Garon Nancy, Bryson Susan E., Smith Isabel M. (2008). Executive Function in Preschoolers: A Review Using an Integrative Framework. Psychological Bulletin.

[B21-jintelligence-10-00125] Gathercole Susan E. (1998). The development of memory. The Journal of Child Psychology and Psychiatry and Allied Disciplines.

[B22-jintelligence-10-00125] Germine Laura, Reinecke Katharina, Chaytor Naomi S. (2019). Digital neuropsychology: Challenges and opportunities at the intersection of science and software. The Clinical Neuropsychologist.

[B23-jintelligence-10-00125] Gerst Elyssa H., Cirino Paul T., Fletcher Jack M., Yoshida Hanako (2015). Cognitive and behavioral rating measures of executive function as predictors of academic outcomes in children. Child Neuropsychology.

[B24-jintelligence-10-00125] Gioia Gerard A., Isquith Peter K., Guy Steven C., Kenworthy Lauren (2000). Behavior rating inventory of executive function: BRIEF.

[B25-jintelligence-10-00125] Golden Charles J. (1978). Stroop Color and Word Test: A Manual for Clinical and Experimental Uses.

[B26-jintelligence-10-00125] Homack Susan, Riccio Cynthia A. (2004). A meta-analysis of the sensitivity and specificity of the Stroop Color and Word Test with children. Archives of Clinical Neuropsychology.

[B27-jintelligence-10-00125] Hughes Claire, Ensor Rosie (2011). Individual differences in growth in executive function across the transition to school predict externalizing and internalizing behaviors and self-perceived academic success at 6 years of age. Journal of Experimental Child Psychology.

[B28-jintelligence-10-00125] Kane Michael J., Engle Randall W. (2003). Working-Memory Capacity and the Control of Attention: The Contributions of Gol Neglect, Response Competition, and Task Set to Stroop Interference. Journal of Experimental Psychology: General.

[B29-jintelligence-10-00125] Kingston Neal M. (2009). Comparability of computer- and paper-administered multiple-choice tests for K-12 populations: A synthesis. Applied Measurement in Education.

[B30-jintelligence-10-00125] Lee Kerry, Bull Rebecca, Ho Ringo M. H. (2013). Developmental changes in executive functioning. Child Development.

[B31-jintelligence-10-00125] Lehto Juhani E., Juujärvi Petri, Kooistra Libbe, Pulkkinen Lea (2003). Dimensions of executive functioning: Evidence from children. British Journal of Developmental Psychology.

[B32-jintelligence-10-00125] Mcmanis Lilla Dale, Gunnewig Susan B. (2012). Finding the Education in Educational Technology with Early Learners. Young Children.

[B33-jintelligence-10-00125] Millisecond Software LLC (2021). Inquisit Web 6.

[B34-jintelligence-10-00125] Miyake Akira, Friedman Naomi P., Emerson Michael J., Witzki Alexander H., Howerter Amy, Wager Tor D. (2000). The Unity and Diversity of Executive Functions and Their Contributions to Complex “Frontal Lobe” Tasks: A Latent Variable Analysis. Cognitive Psychology.

[B35-jintelligence-10-00125] Moran Lisa, Yeates Keith Owen, Kreutzer Jeffrey S., DeLuca John, Caplan Bruce (2018). Stroop Color and Word Test, Children’s Version. Encyclopedia of Clinical Neuropsychology.

[B36-jintelligence-10-00125] Morra Sergio (2015). How do subvocal rehearsal and general attentional resources contribute to verbal short-term memory span?. Frontiers in Psychology.

[B37-jintelligence-10-00125] Muñoz-Sandoval Ana F., Woodcock Richard W., McGrew Kevin S., Mather Nancy (2005). Batería III Woodcock-Muñoz: Pruebas de habilidades cognitivas.

[B38-jintelligence-10-00125] Nelson Hazel E. (1976). A Modified Card Sorting Test Sensitive to Frontal Lobe Defects. Cortex.

[B39-jintelligence-10-00125] Parsey Carolyn. M., Schmitter-Edgecombe Maureen (2013). Applications of technology in neuropsychological assessment. Clinical Neuropsychologist.

[B40-jintelligence-10-00125] Perrotta Carlo, Featherstone Gill, Aston Helen, Houghton Emily (2013). Game-Based Learning: Latest Evidence and Future Directions.

[B41-jintelligence-10-00125] Plaisted James R., Wilkening Greta N., Gustavson John L., Golden Charles J. (1983). The Luria-Nebraska Neuropsychological Battery—Children’s Revision: Theory and Current Research Findings. Journal of Clinical Child Psychology.

[B42-jintelligence-10-00125] Portellano José Antonio, Martínez M. Rosario, Zumárraga Lucía (2011). Evaluación Neropsicológica de las funciones ejecutivas en niños.

[B43-jintelligence-10-00125] Reynolds Cecil R., Kamphaus Randy W. (2015). BASC3: Behavior Assessment System for Children.

[B44-jintelligence-10-00125] Rosas Ricardo, Pizarro Marcelo (2018). WISC-V. Manual de Administración y Corrección.

[B45-jintelligence-10-00125] Rosas Ricardo, Ceric Francisco, Aparicio Andrés, Arango Paulina, Arroyo Rodrigo, Benavente Catalina, Escobar Pablo, Olguín Polín, Pizarro Marcelo, Ramírez María P. (2015). ¿Pruebas tradicionales o evaluación invisible a través del juego? Nuevas fronteras de la evaluación cognitiva. Psykhe.

[B46-jintelligence-10-00125] Rosas Ricardo, Pizarro Marcelo, Grez Olivia, Navarro Valentina, Tapia Dolly, Arancibia Susana, Muñoz-Quezada María Teresa, Lucero Boris, Pérez-Salas Claudia P., Oliva Karen (2022). Estandarización Chilena de la Escala Wechsler de Inteligencia para Niños - Quinta Edición. Psykhe (Santiago).

[B47-jintelligence-10-00125] Rozenblatt Shahal, Kreutzer Jeffrey S., DeLuca John, Caplan Bruce (2018). Stroop Color Word Test (Adult). Encyclopedia of Clinical Neuropsychology.

[B48-jintelligence-10-00125] Rueda M. Rosario, Fan Jin, McCandliss Bruce D., Halparin Jessica D., Gruber Dana B., Lercari Lisha Pappert, Posner Michael I. (2004). Development of attentional networks in childhood. Neuropsychologia.

[B49-jintelligence-10-00125] Schrank Fredrick, McGrew Kevin, Ruef Mary L., Alvarado Criselda G., Muñoz-Sandoval Ana F., Woodcock Richard W. (2005). Overview and Technical Supplement (Batería III Woodcock-Muñoz Assessment Service Bulletin No. 1).

[B50-jintelligence-10-00125] Shing Yee L., Lindenberger Ulman, Diamond Adele, Li Shu-Chen, Davidson Matthew C. (2010). Memory Maintenance and Inhibitory Control Differentiate From Early Childhood to Adolescence. Developmental Neuropsychology.

[B51-jintelligence-10-00125] Soto Elia F., Kofler Michael J., Singh Leah J., Wells Erica L., Irwin Lauren N., Groves Nicole B., Miller Caroline E. (2020). Executive functioning rating scales: Ecologically valid or construct invalid?. Neuropsychology.

[B52-jintelligence-10-00125] Strommen Ellen A. (1973). Verbal Self-Regulation in a Children’s Game: Impulsive Errors on “Simon Says”. Child Development.

[B53-jintelligence-10-00125] Stroop John Ridley (1935). Studies of Interference in Serial Verbal Reactions. Journal of Experimental Psychology.

[B54-jintelligence-10-00125] Subsecretaría de Telecomunicaciones de Chile (2017). IX Encuesta de Acceso y Usos de Internet.

[B55-jintelligence-10-00125] Sweeney Trudy, Geer Ruth (2008). Student capabilities and attitudes towards ICT in the early years (Student capabilities and attitudes towards ICT in the early years). Australian Educational Computing.

[B56-jintelligence-10-00125] Toplak Maggie E., West Richard F., Stanovich Keith E. (2013). Practitioner Review: Do performance-based measures and ratings of executive function assess the same construct?. Journal of Child Psychology and Psychiatry and Allied Disciplines.

[B57-jintelligence-10-00125] United States Census Bureau (2021). Computer and Internet Use in the United States: 2018. https://www.census.gov/newsroom/press-releases/2021/computer-internet-use.html.

[B58-jintelligence-10-00125] Wang Shudong, Jiao Hong, Young Michael J., Brooks Thomas, Olson John (2008). Comparability of computer-based and paper-and-pencil testing in K-12 reading assessments: A meta-analysis of testing mode effects. Educational and Psychological Measurement.

[B59-jintelligence-10-00125] Wiebe Sandra A. (2014). Modeling the emergent executive: Implications for the structure and development of executive function. Monographs Society Res Child.

[B60-jintelligence-10-00125] Wiebe Sandra A., Espy Kimberly Andrews, Charak David (2008). Using confirmatory factor analysis to understand executive control in preschool children: I. Latent structure. Developmental Psychology.

[B61-jintelligence-10-00125] Willoughby Michael T., Blair Clancy B., Wirth R. J., Greenberg Mark (2012). The measurement of executive function at age 5: Psychometric properties and relationship to academic achievement. Psychological Assessment.

[B62-jintelligence-10-00125] Zelazo Philip D., Blair Clancy B., Willoughby Michael T., Larson Meredith, Higgins Erin, Sussman Amy (2016). Executive Function: Implications for Education. https://ies.ed.gov/ncer/pubs/20172000/pdf/20172000.pdf.

[B63-jintelligence-10-00125] Zelazo Philip D., Frye Douglas, Rapus Tanja (1996). An age-related dissociation between knowing rules and using them. Cognitive Development.

[B64-jintelligence-10-00125] Zelazo Philip D., Anderson Jacob E., Richler Jennifer, Wallner-Allen Kathleen, Beaumont Jennifer L., Weintraub Sandra (2013). II. NIH Toolbox Cognition Battery (CB): Measuring executive function and attention. Monographs of the Society for Research in Child Development.

